# Texture Analysis Based on Vascular Ultrasound to Identify the Vulnerable Carotid Plaques

**DOI:** 10.3389/fnins.2022.885209

**Published:** 2022-06-02

**Authors:** Lianlian Zhang, Qi Lyu, Yafang Ding, Chunhong Hu, Pinjing Hui

**Affiliations:** Department of Stroke Center, The First Affiliated Hospital of Soochow University, Suzhou, China

**Keywords:** texture analysis, carotid ultrasound, vulnerable plaques, high-resolution magnetic resonance imaging, atherosclerotic plaque

## Abstract

Vulnerable carotid plaques are closely related to the occurrence of ischemic stroke. Therefore, accurate and rapid identification of the nature of carotid plaques is essential. This study aimed to determine whether texture analysis based on a vascular ultrasound can be applied to identify vulnerable plaques. Data from a total of 150 patients diagnosed with atherosclerotic plaque (AP) by carotid ultrasound (CDU) and high-resolution magnetic resonance imaging (HRMRI) were collected. HRMRI is the *in vivo* reference to assess the nature of AP. MaZda software was used to delineate the region of interest and extract 303 texture features from ultrasonic images of plaques. Following regression analysis using the least absolute shrinkage and selection operator (LASSO) algorithm, the overall cohort was randomized 7:3 into the training (*n* = 105) and testing (*n* = 45) sets. In the training set, the conventional ultrasound model, the texture feature model, and the conventional ultrasound-texture feature combined model were constructed. The testing set was used to validate the model’s effectiveness by calculating the area under the curve (AUC), accuracy, sensitivity, and specificity. Based on the combined model, a nomogram risk prediction model was established, and the consistency index (C-index) and the calibration curve were obtained. In the training and testing sets, the AUC of the prediction performance of the conventional ultrasonic-texture feature combined model was higher than that of the conventional ultrasonic model and the texture feature model. In the training set, the AUC of the combined model was 0.88, while in the testing set, AUC was 0.87. In addition, the C-index results were also favorable (0.89 in the training set and 0.84 in the testing set). Furthermore, the calibration curve was close to the ideal curve, indicating the accuracy of the nomogram. This study proves the performance of vascular ultrasound-based texture analysis in identifying the vulnerable carotid plaques. Texture feature extraction combined with CDU sonogram features can accurately predict the vulnerability of AP.

## Introduction

Ischemic stroke (IS) exhibits a high disability rate, mortality, and recurrence rate, posing a serious threat to human survival and health (Benjamin et al., 2019). Numerous factors influence IS occurrence. In Europe and the United States, previous guidelines for atherosclerosis prevention stratified IS severity according to the degree of carotid stenosis to guide prevention and intervention strategies (Benjamin et al., 2019). However, numerous studies have recently demonstrated that many patients develop IS even with only mild carotid artery stenosis (Cai et al., 2002; Crombag et al., 2019). Therefore, the assessment of IS risk should not be based only on the degree of carotid luminal stenosis, and a comprehensive assessment of the vulnerability of carotid atherosclerotic plaques is required (Yamada et al., 2016).

Atherosclerotic plaque (AP) is a common chronic inflammatory lesion of arterial intima, mainly composed of lipids, inflammatory cells (especially T cells and macrophages), calcium deposition, fibroblasts, and microvessels. The nature of plaques can be categorized into stable and vulnerable. Internal plaque components, such as a thin and incomplete fibrous cap, a large lipid necrotic core, intra-plaque bleeding, intra-plaque neovascularization, and ulcerative plaque formation contribute to plaque vulnerability (Grimm et al., 2013; Yamada et al., 2016; De Havenon et al., 2017; Beg et al., 2020). At present, the commonly used non-invasive and non-radiation imaging methods for evaluating vulnerable plaques are carotid ultrasound (CDU) and cervical high-resolution magnetic resonance imaging (HRMRI). Due to the non-invasive nature, low cost, simplicity of operation, and almost no contraindications, CDU examination is widely employed in clinical practice and has become the first choice for imaging examination. While CDU can distinguish between vulnerable and stable plaques based on their internal echoes and morphology, there are some limitations to its assessment of plaque vulnerability, including the operator’s skill and clinical experience. CDU cannot accurately identify plaque internal components.

High-resolution magnetic resonance imaging is an examination method with high spatial resolution, high tissue resolution, and good reproducibility. Multi-sequence contrast-enhanced imaging can clearly show the substructure of blood vessels from adventitia to the lumen to identify internal carotid plaque components, which is highly consistent with plaque histopathology results (Fairhead et al., 2005; Makris et al., 2011; Brinjikji et al., 2016). However, HRMRI also exhibits limitations in evaluating plaque vulnerability, including its slow imaging speed, many contraindications (such as metal implants or claustrophobia, etc.), and high cost.

With the development of imaging technology and artificial intelligence, new tools have emerged to extract, analyze, and interpret quantitative imaging features, namely radiomics. This process objectively quantifies the change of gray pixel value and potential distribution of lesions (Brinjikji et al., 2016). Texture analysis has recently become a research hotspot in radiomics. This technology can quantitatively analyze the texture features of images in a relatively simple and cost-effectively manner (Lambin et al., 2012; Houssami et al., 2017). These algorithms extract hidden rules from the training dataset and are used for prediction or classification (Tagliafico et al., 2020).

The current imaging omics are mostly based on MRI and CT images and are rarely based on ultrasound images. As far as we know, few studies have been conducted on the differential diagnosis of carotid vulnerable and stable plaques using vascular ultrasound texture analysis combined with LASSO regression. This study aims to determine whether texture analysis based on a vascular ultrasound can be applied to detect plaque vulnerability.

## Materials and Methods

### Study Population

**FIGURE 1:**
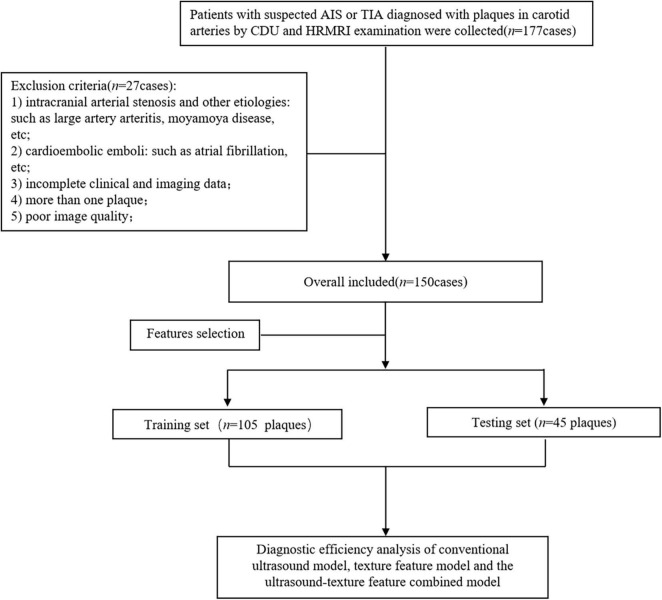
The flowchart of the inclusion and exclusion criteria.

This bidirectional cohort study recruited 150 consecutive patients with suspected acute ischemic stroke (AIS) or transient ischemic attack (TIA), diagnosed with plaques in carotid arteries by CDU and HRMRI examination from the stroke center of the First Affiliated Hospital of Soochow University from May 2016 to December 2020. This study was approved by the Ethics Committee of our organization (No. 2021197). Inclusion criteria: (1) both CDU and HRMRI (Head and carotid plaques) examinations were performed; Exclusion criteria: (1) intracranial arterial stenosis and other etiologies, such as large artery arteritis, moyamoya disease, etc.; (2) cardioembolic emboli, atrial fibrillation, etc.; (3) more than one plaque; (4) incomplete clinical and imaging data; (5) poor image quality (such as unclear wall structure and lumen outline, low signal-to-noise ratio and obvious vascular pulsation artifact). The eligible patients were randomly divided in a 7:3 ratio into the training (*n* = 105) and testing (*n* = 45) sets. The flow chart displays the analysis path of this study (Figure 1).

### Carotid Ultrasound Protocol

All patients were diagnosed using the iU Elite scanner (Phillips Medical System, Holland). The L9-3 linear array probe and C5-1 convex array probe were used to assess the blood vessels from the proximal to the distal segment. Continuous cross-sectional and longitudinal scans were performed. The bilateral common carotid arteries (CCA), carotid artery bulb (CAB), and internal carotid arteries (ICA) were observed by gray scale imaging, color flow imaging, and spectral Doppler analysis. If plaques were observed, the plaque size, shape, echo, integrity, and degree of vascular stenosis were determined using multi-section and multi-angle imaging, as well as the peak systolic velocity, the end-diastolic velocity, and the resistance index (Lyu et al., 2021a). All the images of carotid atherosclerotic plaques were saved and analyzed.

### Carotid Ultrasound Analysis

The CDU criteria to evaluate the nature of atherosclerotic plaques (Johnsen and Mathiesen, 2009; Ten Kate et al., 2013; Jang et al., 2020) are described below. Vulnerable plaques are heterogeneous, hypoechoic, or moderately hypoechoic, with or without plaque surface shape irregularity or incomplete fibrous cap or plaque blood flow signal (ulcerative plaque). Stable plaques are homogeneous with moderate or strong echo, with a regular plaque surface and intact fibrous cap. Two observers independently evaluated the images.

### High-Resolution Magnetic Resonance Imaging Protocol

All patients underwent an HRMRI examination using the GE Signa HDXT 3.0T (GE Healthcare System, United States) MRI scanner, selecting the head and neck joint coil, ECG gating, and the standard carotid artery multi-sequence contrast imaging scheme. A 2D time of flight MR angiography (TOF MRA) imaging was performed to determine the location of the bifurcation of the common carotid artery. After locating the common carotid artery bifurcation, a 3DTOF MRA imaging was performed, followed by rotational imaging reconstruction using maximum intensity projection (MIP). The plaque location was determined by combining the cross-sectional position of TOF and the reconstructed image. High-resolution target scanning with black-blood sequence (including T1WI, T2WI, and PDWI) was performed on cervical vessels and targeted plaques. The parameters were as follows. Routine DWI scans of the head: 5400.00 ms repetition time (TR)/75.20 ms echo time (TE); 220.0 mm × 220.0 mm field of view (FOV); 2, number of excitation (NEX); 256 × 256 matrix; 41 s total scanning time. 3D TOF MRA: 15.00 ms TR/3.45 ms TE; 200.0 mm × 200.0 mm FOV; 2 NEX; 512 × 512 matrix; 4 min 47 s total scanning time. T1WI: 500.00 ms TR/9.82 ms TE; 160 × 160 mm FOV; 4 NEX; 512 × 512 matrix; 5 min scanning time. T2WI: 3,560.00 ms TR/71.04 ms TE; 160.0 mm × 160.0 mm FOV; 6 NEX; 512 × 512 matrix, 3 min 51 s scanning time. PDWI: 1980.00 ms TR/11.30 ms TE, 160.0 × 160.0 mm FOV, 2 min 50 s scanning time. Both Gd-DTPA enhanced T1WI and Gd-DTPA were injected into the cubital vein at a concentration of 0.1 mmol/kg. All HRMRI data were measured and calculated using Siemens syngo MR B15 software.

### High-Resolution Magnetic Resonance Imaging Analysis

The American Heart Association (AHA) classification was used for MRI analysis (Lubner et al., 2017): for vulnerable plaques, type IV-V: plaques with large lipid necrotic core and fibrous caps with a small amount of calcification and type VI: plaque surface ulcers, or intra-plaque bleeding and thrombosis and for stable plaques, type III: diffuse intimal thickening or small non-calcified eccentric plaques; type VII: calcified plaque; and type VIII: fibrous plaque without a fat nucleus, with a small amount of calcification (Supplementary Figure 1). Previous studies have demonstrated that HRMRI results are highly consistent with plaque histopathology in evaluating plaque vulnerability, so this study uses HRMRI results as the *in vivo* reference to assess plaque properties (Fairhead et al., 2005; Makris et al., 2011; Brinjikji et al., 2016). Two observers independently evaluated the images.

### Texture Protocol

#### Patch Image Segmentation

**FIGURE 2:**
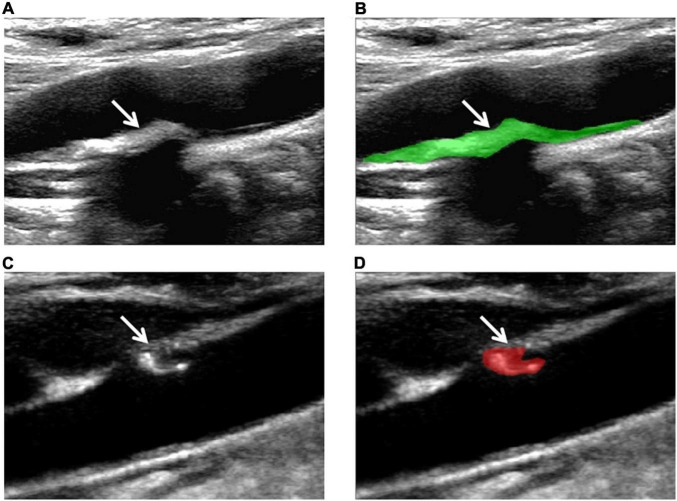
Examples of the manual segmentation in stable and vulnerable plaques. **(A)** Original ultrasound image of stable plaque (arrow). **(B)** The segmented area was within the green contour on the largest area on stable plaque (arrow). **(C)** Original ultrasound image of vulnerable plaque (arrow). **(D)** The segmented area was within the red contour of the largest area on the vulnerable plaque (arrow).

The ultrasonic images of 150 plaques were acquired from the institution’s PACS in BMP format. The region of interest (ROI) was manually drawn using MaZda software (version 4.6.0, Institute of Electronics, Rhodes University of Technology). According to HRMRI results, the following steps were applied: ROI was manually determined along the maximum area of the plaque on the CDU longitudinal section and the arbitrary shape of the CDU plaque was drawn with two different colors. Figure 2 illustrates the region of interest on the CDU image of carotid plaque.

#### Texture Feature Extraction

In order to avoid interference caused by insufficient image contrast (uneven brightness distribution of image pixels), the ultrasonic images of 150 plaques were first normalized by MATLAB R2020 so that the gray value of pixels was distributed between 0 and 1. Each ROI was delineated by two experienced sonographers, both blinded to the actual nature of the plaque. Then, gray-scale normalization was performed between m ± 3d (where m represented the mean value of the gray levels within the ROI; d represented the standard deviation) to reduce the influence of data acquisition environment, acquisition parameters, and other factors on a gray scale image. This step improves the comparability and reliability of the experimental results, as applied in previous studies (Galm et al., 2018; Oh et al., 2019). 303 texture features were extracted from each region of interest based on 6 texture feature algorithms: gray scale histogram (Histogram), gray absolute gradient (Absolute gradient), run matrix (Run-length matrix), gray co-occurrence matrix (Gray-level co-occurrence matrix), autoregressive model (Autoregressive model), and wavelet analysis (Wavelet transform). A detailed description of these texture features can be found on the official MaZda website.^1^

#### Texture Feature Selection

Subsequently, another experienced sonographer segmented the regions of interest independently from 50 randomly selected plaques to evaluate the stability and repeatability of these texture features. All sonographers were blinded to the actual nature of the plaques. The intraclass correlation coefficient (ICC) values of each texture feature were calculated. Only features with an ICC value of ≥0.75 were considered repeatable and selected for further analysis (Khan et al., 2015). After ICC screening, 238 different texture features were selected for the following process. The least absolute shrinkage and selection operator (LASSO) algorithm was applied to minimize the potential collinearity of variables measured from the same patient and the overfitting of variables. For multivariable analyses, the L1-penalized least absolute shrinkage and selection algorithm augmented with 10-fold cross-validation was used for internal validation. This logistic regression model penalizes the absolute size of the regression model’s coefficients based on the value of λ. The penalty determines the rate at which the weak factors shrink toward zero, retaining only the strongest predictors in the model. The most predictive plaque indicators were selected by the minimum λ. The R package “glmnet” statistical software (R Foundation) was employed to perform the LASSO algorithm.

#### Nomogram Construction and Validation

The classification and Regression Training package in R version 3.6.1 was used to divide patients into a training set (105 plaques) and a testing set (45 plaques). The construction of the nomogram was based on the analysis in the training set. The results of multivariate logistic regression were further used to formulate the nomogram by applying the rms package in R version 3.6.1. Two criteria, the consistency index (C-index) and the calibration curve, were used to validate the nomogram model in the testing set. The C-index with a value range between 0 and 1, was used to evaluate the performance of the model. A larger C-index (>0.70) indicates better performance of the model. A calibration curve close to the ideal one was considered to indicate the accuracy of the nomogram prediction.

### Statistical Analysis

Based on the HRMRI evaluation results, the patients were divided into stable and vulnerable plaque groups. The clinical and imaging data were analyzed by the chi-square test, the *t*-test, and the Mann–Whitney U test. In this study, three logistic regression models have been established: the conventional ultrasonic logistic regression model, the texture feature logistic regression model, and the conventional ultrasonic-texture feature logistic regression combined model. The variables included in the final multiple logistic regression were determined by stepwise regression. The areas under the receiving operating characteristic (ROC) curves were used to evaluate the accuracy of the three models in identifying the nature of carotid plaques. The area under the curve (AUC), sensitivity, and specificity were represented in a graphical plot. The interobserver agreement was calculated using Cohen’s Kappa statistics or ICC values assessed by observer A and observer B. All tests were two-tailed, with a *p*-value threshold of 0.05 for statistical significance. Statistical analyses were conducted in R statistical software (version 3.6.1).^2^

## Results

### Comparison of Baseline Data Between the Two Groups

This study included 150 consecutive patients (mean age, 61.7 ± 10.0 years; 120 [80.0%] men); 116 (77.3%) had hypertension, 53 (36.3%) had diabetes, and 51 (34.0%) had coronary heart diseases. The patient characteristics are listed in Table 1, and there were no significant differences between the vulnerable plaques group and the stable plaques group in terms of age, gender, hypertension, diabetes, coronary heart diseases, history of alcohol intake, current or former smokers, and laboratory tests.

**TABLE 1:** Baseline data of patients in the stable plaque group and vulnerable plaque group (*n* = 150).

Subject characteristic	Stable plaques group (*n* = 57)	Vulnerable plaques group (*n* = 93)	*P*-value
Age (median ± SD) (year)	62.9 ± 9.3	61.0 ± 10.4	0.277
Male(n) (%)	45 (78.9)	75 (80.6)	0.605
Hypertension(n) (%)	34 (59.6)	75 (80.6)	0.005
Systolic blood pressure (median ± SD) (mm Hg)	138.8 ± 18.8	140.8 ± 18.3	0.520
Diastolic blood pressure (median ± SD) (mm Hg)	81.6 ± 10.4	81.2 ± 12.2	0.855
Diabetes(n) (%)	23 (40.4)	30 (32.3)	0.314
Blood glucose (median ± SD) (mmol/L)	5.7 ± 1.5	5.6 ± 1.6	0.699
Coronary heart disease (n) (%)	21 (36.8)	30 (32.3)	0.565
Dyslipidemia(n) (%)	13 (22.8)	26 (28.0)	0.485
TC (median ± SD) (mmol/L)	3.9 ± 1.1	4.0 ± 1.1	0.499
TG (median ± SD) (mmol/L)	1.5 ± 0.7	1.5 ± 0.6	0.495
HDL-C (median ± SD) (mmol/L)	1.2 ± 0.5	1.1 ± 0.5	0.547
LDL-C [Median (Q1 - Q3)] (mmol/L)	2.2[1.7–2.8]	2.2[1.7–3.0]	0.827
hs-CRP [Median (Q1 - Q3)] (mmol/L)	3.6[2.3–6.8]	3.5[2.2–6.7]	0.763
Uric acid (median ± SD) (mmol/L)	315.2 ± 87.2	290.7 ± 93.5	0.112
Fibrinogen [Median (Q1 - Q3)] (g/L)	2.2[1.7–2.8]	2.2[1.7–3.0]	0.207
HCY (median ± SD) (mmol/L)	8.5 ± 3.7	8.0 ± 3.5	0.385
Neurologic symptoms			
Unilateral limb symptoms (n) (%)	32 (56.1)	48 (51.6)	0.445
Indistinct speech (n) (%)	18 (31.6)	24 (25.8)	0.784
Blurred vision (n) (%)	14 (24.6)	12 (12.9)	0.067
Dizzy (n) (%)	18 (31.6)	25 (28.9)	0.537
TIA (n) (%)	9 (15.8)	15 (16.1)	0.956
History of alcohol intake (n) (%)	21 (30.6)	35 (37.6)	0.922
Current or former smokers (n) (%)	11 (19.3)	28 (30.1)	0.143

*Numbers are given as n (%) or mean ± SD or median (Q1–Q3). TC, total cholesterol; TG, triglycerides; HDL-C, high-density lipoprotein cholesterol; LDL-C, low-density lipoprotein cholesterol; hs-CRP, high-sensitivity C-reactive protein; HCY, homocysteine; TIA, transient ischemic attack.*

### Efficiency of Conventional Ultrasonic Model

The diagnostic model was constructed with four variables of conventional ultrasound: surface morphology, fibrous cap state, plaque echo, and plaque ulcer formation. In the training set, 83 vulnerable and stable plaques were accurately identified. The accuracy, sensitivity, and specificity of the model were 79.05, 85.94, and 68.29%, respectively. In the testing set, 35 vulnerable and stable plaques were accurately identified, resulting in an accuracy, sensitivity, and specificity of 77.78, 75.86, and 81.25%, respectively.

### Feature Extraction and Efficiency of Texture Feature Model

**FIGURE 3:**
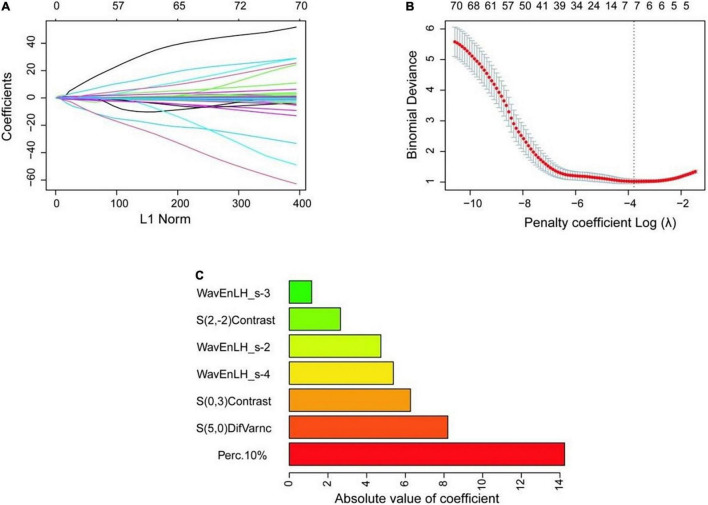
Selection process of texture features. **(A)** The variation of coefficient of the variable with penalty coefficient. **(B)** The optimal penalty coefficient is selected by 10-fold cross-validation, and seven optimal texture features are selected when the binomial deviation is the smallest (minimum standard). **(C)** The absolute value of the coefficient of variables is finally included.

To seek potential significant texture features, we identified the nature of carotid plaques thought non-zero coefficients in the logistic regression model (Figure 3). Only seven features were selected for the texture feature model (Perc.10*%*, WavEnLH_s-2, WavEnLH_s-3, WavEnLH_s-4, S(2,-2)Contrast, S(0,3)Contrast, S(5,0)DifVarnc) (Table 2). In the training set, 86 vulnerable and stable plaques were accurately identified, yielding an accuracy, sensitivity, and specificity of 81.90, 87.50, and 73.17%, respectively. In the testing set, 36 vulnerable and stable plaques were accurately identified. The accuracy, sensitivity, and specificity of the model were 80.00, 72.41, and 93.75%, respectively. All were higher compared with the conventional ultrasound model (except for sensitivity in the testing set).

**TABLE 2:** Statistically significant texture features extracted from 150 carotid plaques and conventional ultrasonic characteristics.

Feature	Stable plaques group (*n* = 57)	Vulnerable plaques group (*n* = 93)	*P*-value
**Histogram**			
Perc.10%	41.0 ± 26.9	23.2 ± 16.2	<0.001
**Co-occurrence matrix**			
S(2,-2)Contrast	63.9 ± 33.5	36.8 ± 21.6	<0.001
S(0,3)Contrast	97.8 ± 47.7	57.7 ± 28.9	<0.001
S(5,0)DifVarnc	16.8 ± 9.5	15.6 ± 11.3	0.03
**Wavelet**			
WavEnLH_s-2	267.9 ± 181.6	128.5 ± 83.2	<0.001
WavEnLH_s-3	535.1 ± 378.9	248.8 ± 138.9	<0.001
WavEnLH_s-4	721.9 ± 447.4	401.8 ± 200.6	<0.001
**Conventional ultrasonic variables(n) (%)**			
Surface morphology			<0.05
Regular	56 (37.3)	26 (17.3)	
Irregular	1 (0.6)	67 (44.7)	
Fibrous cap state			<0.05
Intact	55 (36.7)	68 (45.3)	
Crippled	2 (1.3)	25 (16.7)	
Hypoechoic/mainly Hypoechoic plaque			0.02
Yes	49 (32.7)	61 (40.7)	
No	8 (5.3)	32 (21.3)	
Ulcerative plaque			0.02
Yes	56 (37.3)	85 (56.7)	
No	1 (0.6)	8 (5.3)	

### Efficiency of the Conventional Ultrasound-Texture Feature Combined Model

Finally, the combined model was constructed using eight variables (Perc.10*%*, WavEnLH_s-2, WavEnLH_s-3, WavEnLH_s-4, S(2,-2)Contrast, S(5,0)DifVarnc, surface morphology, and fibrous cap state). In the training set, 89 vulnerable and stable plaques were accurately identified. The accuracy, sensitivity, and specificity of the model were 84.76, 77.94, and 97.30%, respectively. In the testing set, 39 vulnerable and stable plaques were accurately identified, yielding an accuracy, sensitivity, and specificity of 86.67, 84.00, and 90.00%, respectively.

### Comparison of the Diagnostic Performance of Different Models

**FIGURE 4:**
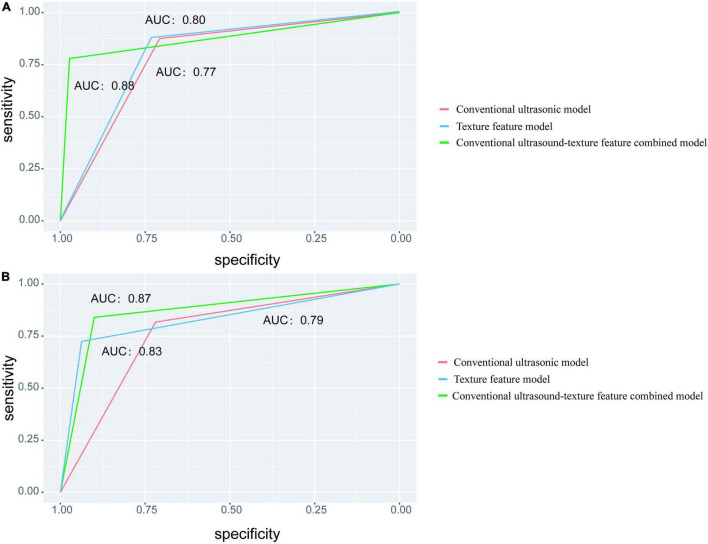
Different models for predicting classification performance. **(A)** The ROC curve with the AUC value for the training set. **(B)** The ROC curve with the AUC values for the testing set.

In the training set, the highest AUC (0.88), accuracy (84.76%), and specificity (97.30*%)* were observed in the combined model, whereas the highest sensitivity (87.50*%*) was found in the texture feature model. In the testing set, the highest AUC (0.87), accuracy (86.67*%)*, and sensitivity (90.00*%)* were found in the combined model, while the specificity of the texture feature model was the highest among the models (Figure 4). Statistical studies revealed that the combined model had the highest AUC in both the training and testing sets, with a statistically significant difference.

### Construction and Validation of the Nomogram

**FIGURE 5:**
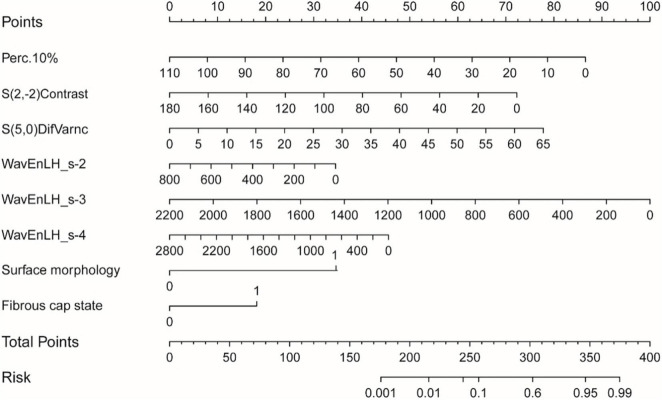
The nomogram was constructed based on the combined model, and the scores of each variable were added to obtain the total score, corresponding to the risk probability of predicting a vulnerable plaque. The nature of each plaque in the nomogram can be directly read out.

According to the final combined model variables, a nomogram containing independent risk factors was established. The scores of items displayed in the nomogram should be added together. As illustrated in Figure 5, WavEnLH_s-3 contributed most to risk, followed by Perc.10%, S(5,0)DifVarnc, and S(2,-2)Contrast. C-index and calibration curves were utilized to validate the nomogram’s predictive accuracy. The C-indexes observed in the training (0.89) and testing sets (0.84), indicated that this model is accurate. Furthermore, the ideal and calibration curves were close in the testing set (Supplementary Figure 2). These results revealed that the nomogram model possessed a high degree of discrimination.

### Interobserver Agreement

The Cohen’s kappa between two observers for evaluating plaque vulnerability by CDU was 0.95 (95% CI = 0.88 to 0.98), 0.89 (95% CI = 0.82 to 0.96) for the surface morphology of plaque, 0.88 (95% CI = 0.78 to 0.97) for the fibrous cap state, 0.89 (95% CI = 0.81 to 0.97) for hypoechoic/mainly hypoechoic plaque, and 0.71(95% CI = 0.49 to 0.93) for ulcerative plaque; The Cohen’s kappa between two observers for evaluating plaque vulnerability by HRMRI was 0.94 (95% CI = 0.89 to 0.99), 0.90 (95% CI = 0.86 to 0.97) for the surface morphology of plaque, 0.92 (95% CI = 0.86 to 0.99) for the fibrous cap state, 0.92 (95% CI = 0.85 to 0.99) for intra-plaque hemorrhage, 0.90 (95% CI = 0.84 to 0.95) for lipid necrotic core, and 0.88 (95% CI = 0.75 to 0.96) for ulcerative plaque.

## Discussion

In this study, a diagnosis model based on vascular ultrasound combined with a LASSO algorithm was constructed to identify vulnerable and stable carotid plaques. The conventional ultrasound-texture feature combined model demonstrated satisfactory diagnostic performance.

The vulnerability of carotid plaque is affected by multiple risk factors. These pathological processes interact with each other, greatly increasing their pathogenic effects. Previous studies have confirmed that age, gender, diabetes mellitus, hyperlipidemia, smoking, and other factors are closely related to the development of atherosclerosis (Rubinat et al., 2016; Lyu et al., 2021b). In this study, there was no significant difference between patients from the stable and the vulnerable groups in terms of age, gender, diabetes mellitus, coronary heart diseases, history of alcohol intake, current or former smokers, and laboratory tests. Study bias may arise from the sample size, plaque location, study methods, and underlying diseases. Izzo et al. (2015) investigated 2,143 patients with hypertension with a follow-up of 56.6 months, revealing that about 1/3 had new-onset carotid plaque formation. Marfella et al. (2007) observed that patients with hypertension with peak blood pressure in the morning had more inflammatory components and poor stability within carotid plaques. Long-term hypertension leads to intimal thickening and loss of elasticity, especially in the presence of elevated systolic blood pressure. In addition, mechanical stress, sympathetic nerve activity, and vasoconstrictor levels are increased. This causes intimal damage and promotes lipid deposition, platelet adhesion, and aggregation, gradually forming vulnerable plaques. Therefore, carotid plaques tend to be unstable in patients with hypertension. Moreover, systolic blood pressure plays a greater role in the process of transformation of plaque vulnerability, and the results of our study are in accordance with previous studies (Fusegawa et al., 2006; Iwata et al., 2012). Minimizing risk factors, accurately and timely identifying carotid plaque vulnerability, and adopting effective interventions are important approaches to preventing acute cerebrovascular events.

The plaque’s nature is determined by its internal components (Lubner et al., 2017). At present, gray scale ultrasound is commonly used in clinical practice and has gradually become a screening method; however, differential diagnosis using gray scale ultrasound is somewhat subjective to the operator’s skill and clinical experience. Plaque echo varies according to its internal composition. Although plaque properties can be identified by their echoes, they cannot accurately distinguish their internal components (such as intra-plaque bleeding and lipid core). Furthermore, they are easily affected by calcified sound and shadow, respiratory movement, and operator skill, and they are insensitive to plaque surface structural features such as fibrous caps and ulcers (Kwee et al., 2009).

With the development of imaging technology and artificial intelligence, radiomics extracts and quantifies the texture features of medical images, significantly reducing the variability of the personal experience of radiologists (Lambin et al., 2012). In the past, researchers attempted to carry out research on related aspects such as lesion detection, tumor prognosis, or efficacy evaluation for multiple organs and multiple diseases in order to explore new ways of disease diagnosis and treatment by texture analysis technology (Friedrich-Rust et al., 2009; Anderson et al., 2012). However, most of them were based on imaging examinations such as CT and MRI, and relatively few were based on ultrasonic images. In particular, for the vulnerability of carotid plaques, our study explored the diagnostic value of texture analysis technology based on ultrasonic images of carotid plaques.

The size, shape, surface irregularity, complex composition, rich lipids, calcification, and other information of plaques affect each texture’s eigenvalue (Baeßler et al., 2018). In a previous study (Rakebrandt et al., 2000), the histopathology of 10 carotid plaques after carotid endarterectomy revealed that five texture features corresponded to the plaque tissue components (fibrin, elastin, calcium, bleeding, and lipid). Since 2010, multiple studies have explored texture features from ultrasound images of plaques to describe their properties (Acharya et al., 2012; Zhou et al., 2019). Acharya et al. (2012) presented a local binary pattern (LBP)/law’s texture energy (LTE) technique based on two carotid datasets for ultrasound characterization of plaques, picking each significant combination of texture features. When using the atheromatic-based system on semiautomatically determined plaque regions, the support vector machine (SVM) classifier was adapted with the highest accuracy of 83%. In our study, eight statistically significant features were extracted from the ultrasound images of carotid plaques, including six textural features and two morphological features, and an accuracy rate of 84.76% was obtained in the evaluation of vulnerable plaques. Our findings indicated that the combination of textural and morphological features may improve the classification ability of plaque properties. Azzopardi et al. (2020) extracted phase maps from two-dimensional (2D) carotid ultrasound images as input to convolutional neural networks (CNNs) to segment the carotid media-adventitia boundary (MAB) with SegNet. However, the full set of images used in that study was derived from only five patients, resulting in a large correlation of training and testing data, the validity of which requires a larger size. van Engelen et al. (2014) extended texture features to three-dimensional (3D) ultrasound images of carotid arteries. In total, 298 patients with carotid atherosclerosis were evaluated at baseline and after 1 year; carotid plaque volume and 376 measures of plaque texture were assessed. The study showed that combined changes in texture and total plaque volume provided the best predictor of vascular events. This study demonstrates that texture analysis based on 3D ultrasound holds great clinical value, which provides a direction for our next research.

The AUC and accuracy of the combined model were higher than that of the conventional ultrasonic model and the texture feature model in the training and testing sets, indicating the predictive value of the combined model. In the training set, the conventional ultrasonic model, the texture feature model, and the combined model training set’s AUC values were 0.77, 0.80, and 0.88, respectively; and in the testing set, the AUC values were 0.79, 0.83, and 0.87, respectively. The performance of the combined model was slightly lower, but the AUC was >0.85, indicating the reliability and stability of the model, without obvious overfitting. The LASSO algorithm is a compression estimate with extremely low data requirements and wide application. It gets a more refined model by constructing a penalty function, which compresses some coefficients, sets some smaller coefficients, and even directly changes some coefficients with smaller absolute values to 0. In addition, LASSO can filter the variables, reduce the complexity of the model, and make the model relatively stable. Unlike traditional statistical analysis, the combination of LASSO algorithm and logistic regression in this study greatly improves the efficiency of differential diagnosis between stable and vulnerable plaques and provides a future method for objective differential diagnosis in clinical practice.

This research has several limitations. First, this is a bidirectional cohort study, so selection bias cannot be completely avoided. Second, the evaluation of plaque properties by CDU and HRMRI and the manual delineation of ROI involved some bias. Third, the number of cases in this study is relatively small, and the sample size should be expanded in the future. Moreover, this study only established a model based on imaging, and a future model that combines clinical information with imaging may improve the efficiency. Finally, not all lesions can be confirmed pathologically in clinical practice. However, we believe that a similar diagnostic efficiency can be achieved by combining various techniques to assess the pathological findings.

## Conclusion

This research proves the satisfactory performance of vascular ultrasound-based texture analysis in identifying vulnerable carotid plaques. Therefore, texture feature extraction in conjunction with CDU sonogram features can accurately predict the properties of atherosclerotic plaques.

## Data Availability Statement

The raw data supporting the conclusions of this article will be made available by the authors, without undue reservation.

## Ethics Statement

The studies involving human participants were reviewed and approved by the Ethics Committee of the First Affiliated Hospital of Soochow University (No. 2021197). Written informed consent was obtained from the individual(s) for the publication of any potentially identifiable images or data included in this article.

## Author Contributions

PH: guarantor of the article. LZ and QL: conception and design of the idea and collection and assembly of data. YD and CH: data analysis and interpretation. All authors contributed to the article and approved the submitted version.

## Conflict of Interest

The authors declare that the research was conducted in the absence of any commercial or financial relationships that could be construed as a potential conflict of interest.

## Publisher’s Note

All claims expressed in this article are solely those of the authors and do not necessarily represent those of their affiliated organizations, or those of the publisher, the editors and the reviewers. Any product that may be evaluated in this article, or claim that may be made by its manufacturer, is not guaranteed or endorsed by the publisher.

## Footnotes

1
http://www.eletel.p.lodz.pl/programy/mazda/

2
http://www.Rproject.org

## Supplementary Material

Supplementary Figure 1 Corresponding multi-contrast magnetic resonance (MR) images of two typical carotid plaques in the lateral imaging view. **(A,E)** HRMRI T1WI images. **(B,F)** HRMRI T1WI C + images. **(C,G)** HRMRI T2WI images. **(D,H)** HRMRI PDWI images. The first plaque (first row, **A–D**) was obtained from the distal segment of the left common carotid artery extending to the anterior wall of the internal carotid artery of a 62-year-old patient. The presence of moderate enhancement, defined as neovascularization or inflammation in the T1WI C + (indicated by the white arrow) and the lipid necrotic core in the T2WI (indicated by the white arrow), indicates the plaque is more likely to be vulnerable; The second plaque **(E–H)** was acquired from the posterior wall of left internal carotid artery of a 70-year-old patient. The fibrillar component is observed as the high-intensity area in the T2WI and PDWI (indicated by the white arrow); the high risk composition was absent, indicating the stability of this plaque.

Supplementary Figure 2 **(A)** The calibration curve of the nomogram model. Apparent represents the original curve, Ideal represents the reference line obtained by the ideal model, and Bias-corrected represents the calibration curve. The ideal curves and calibration curves were very close in the testing set. **(B)** DCA curve for the nomogram. The net benefit was plotted vs. the threshold probability. The dotted line represents the nomogram. The gray and black lines represent the treat-all-patients scheme or the treat-none scheme, respectively. DCA, decision curve analysis.
