# Supplementary figures and images for: Texture Analysis Based on Vascular Ultrasound to Identify the Vulnerable Carotid Plaques
Source: Front Neurosci. 2022 Jun 2;16:885209. doi: 10.3389/fnins.2022.885209 (PMC9204477; doi:10.3389/fnins.2022.885209)

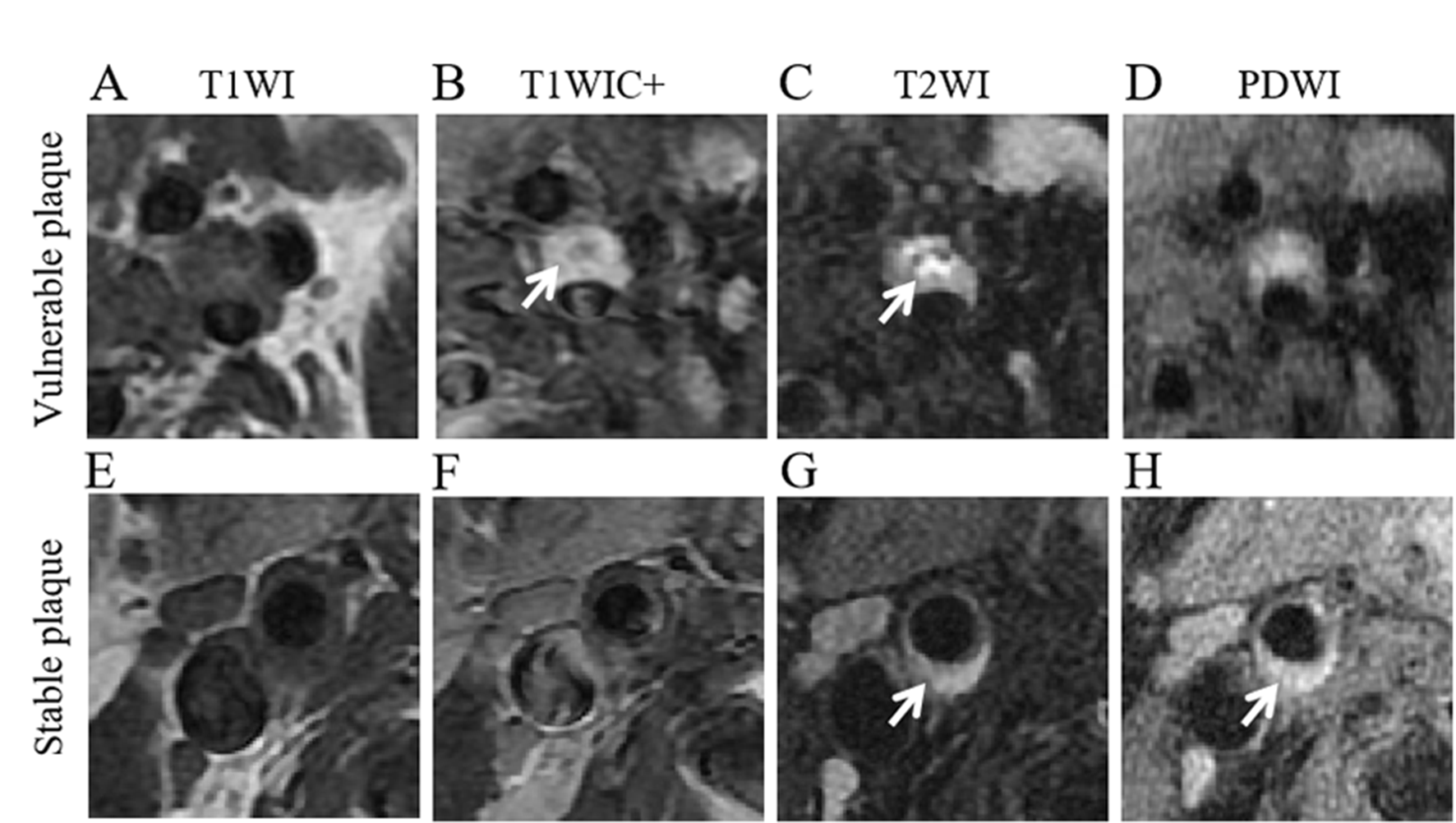

Supplement: Supplementary Figure 1 — Corresponding multi-contrast magnetic resonance (MR) images of two typical carotid plaques in the lateral imaging view. (A,E) HRMRI T1WI images. (B,F) HRMRI T1WI C + images. (C,G) HRMRI T2WI images. (D,H) HRMRI PDWI images. The first plaque (first row, A–D) was obtained from the distal segment of the left common carotid artery extending to the anterior wall of the internal carotid artery of a 62-year-old patient. The presence of moderate enhancement, defined as neovascularization or inflammation in the T1WI C + (indicated by the white arrow) and the lipid necrotic core in the T2WI (indicated by the white arrow), indicates the plaque is more likely to be vulnerable; The second plaque (E–H) was acquired from the posterior wall of left internal carotid artery of a 70-year-old patient. The fibrillar component is observed as the high-intensity area in the T2WI and PDWI (indicated by the white arrow); the high risk composition was absent, indicating the stability of this plaque. [file Image_1.TIF]

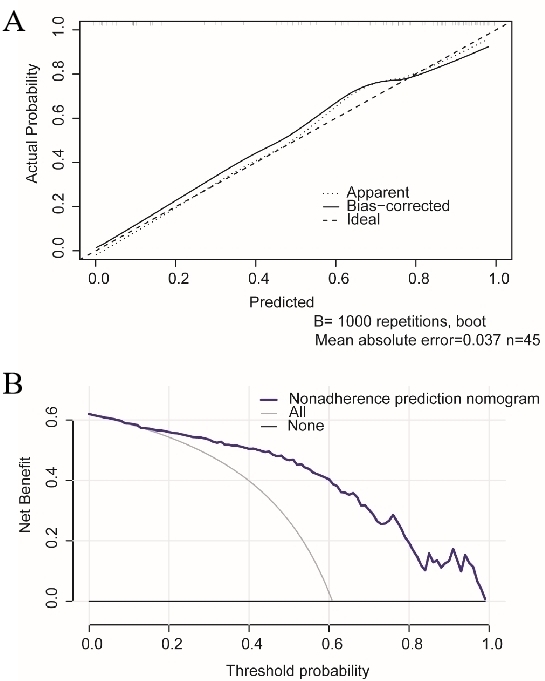

Supplement: Supplementary Figure 2 — (A) The calibration curve of the nomogram model. Apparent represents the original curve, Ideal represents the reference line obtained by the ideal model, and Bias-corrected represents the calibration curve. The ideal curves and calibration curves were very close in the testing set. (B) DCA curve for the nomogram. The net benefit was plotted vs. the threshold probability. The dotted line represents the nomogram. The gray and black lines represent the treat-all-patients scheme or the treat-none scheme, respectively. DCA, decision curve analysis. [file Image_2.JPEG]
